# New Drugs, Appliances, and Things Medical

**Published:** 1892-07-16

**Authors:** 


### NEW DRUGS, APPLIANCES, AND THINGS MEDICAL.

[All preparations, appliances, novelties, etc., of which a notice is desired, should be sent for The Editor, to care of The Manager, 140, Strand, London, W.C.]

### "ST. BEDE" DISINFECTANT.

(THE ST. BEDE CHEMICAL COMPANY, Newcastle-on-Tyne.)

This is stated to contain 4 per cent. of mercuric chloride with acid sodic sulphate, a proportion of eucalyptus and thymol, and to be coloured with indigo. It is put up in small bricks. One of these dissolved in a quart of water forms a 4 per cent. solution of the mercury salt. All the above ingredients were easily discovered by analysis. The block is freely soluble in hot water, but takes some little time to dissolve in cold. It is well established that mercuric chloride is the most powerful antiseptic known, consequently we have here a convenient and useful form in which to use it. The only drawback to its use is its very poisonous nature, but the public is duly warned by labels fixed to each parcel. The experiments detailed in the accompanying pamphlet show conclusively the strong germ-destroying power of the "St. Bede" Disinfectant.

### THE RELIANCE MEDICINE OR LIQUID DROPPER.

(THE DRUGGISTS' SPECIALITY CO., 44, Stockorchard Road, N.)

This consists of two right-angled glass tubes fitting into a cork which can be placed in any bottle from which drops are required to be taken. To use it, place the dropper in a bottle as you would an ordinary cork, turn one of the tubes till the apertures of both tubes are opposite to each other, pour through the larger tube and press the end of the fingers on the aperture of the smaller tube. The dropping will increase or diminish according to the pressure given. We have tried the specimen forwarded to us; it answered well, and the apparatus seems to us to cheaply fill a want. The only wonder is that it has not been thought of before.

### TRITICUMINA WHOLE MEAL BREAD.

To those who are unacquainted with Triticumina Bread we can most confidently recommend it as the most palatable whole meal bread we have tasted. Its claims to special nutritive qualities are confirmed by a large number of experts on the subject of food. The process used in its preparation causes a large portion of the starch to be converted into maltose and dextine. The digestive qualities of the bread are increased by the grinding which the grain undergoes. The triticumina factory is in Reading, but agents are to be found in all parts of London and the country.

### KOPS' ALE.

This is one of the latest, and, as far as our experience goes, one of the best temperance drinks yet invented. In appearance and also in taste it much resembles mild ale; but the makers warrant it to be non-alcoholic and pure. It possesses considerable "sparkle" and is a most refreshing beverage, having none of that disagreeable sweetness so often found in teetotal compounds. One of the specimens we tried had been upwards of a year in bottle and another was only a few weeks old. The older specimen had lost none of its sharpness and was quite equal to the new. The ale, therefore, has the advantage that it may be kept with perfect safety. It is manufactured at the Kops' Brewery, Wandsworth Bridge Road, Fulham, and is sold in imperial half-pints and pints. The stoppers are perfectly air-tight and easily removable.

